# A machine learning framework to identify complex physicochemical features of B cell epitopes

**DOI:** 10.21203/rs.3.rs-6255613/v1

**Published:** 2025-04-18

**Authors:** Simranjit Grewal, Uwa Iyamu, Daniel Vinals, Catherine Mitran, Nidhi Hegde, Stephanie Yanow

**Affiliations:** University of Alberta; University of Alberta; University of Alberta; University of Alberta; University of Alberta; University of Alberta

## Abstract

During infection with *Plasmodium falciparum* in pregnancy, parasites express a unique virulence factor, VAR2CSA, that mediates binding of infected red blood cells to the placenta. A major goal in designing vaccines to protect pregnant women from malaria is to elicit antibodies to VAR2CSA. The challenge is that VAR2CSA is highly polymorphic and identifying conserved epitopes is essential to elicit strain-transcending immunity. Unexpectedly, a mouse monoclonal antibody, 3D10, raised against the unrelated Duffy binding protein from *P. vivax* (DBPII) cross-reacts with diverse alleles of VAR2CSA *in vitro*. To identify these potentially conserved epitopes in VAR2CSA, we designed a machine learning framework to analyse 3D10 reactivity to peptides derived from two alleles of VAR2CSA, DBPII, and PvEBP2 (negative control). We used decision trees and a panel of 430 features to extract features correlated to 3D10 binding. We analysed patterns of these features in the dataset and designed mutant peptides to test complex sequence motifs. Features associated with 3D10 reactivity were mapped onto predicted 3D structures of *Plasmodium* proteins and validated based on 3D10 reactivity to the recombinant antigens. While the array data identified certain linear epitopes, the framework predicted other epitopes that are conformational. With this approach, peptide array data can be mined to extract physicochemical properties of epitopes recognized by polyreactive antibodies.

## 2. Introduction

Malaria caused by *Plasmodium falciparum* is among the deadliest infectious diseases and results in more than half a million deaths per year^1^. Pregnant women compose a large portion of the at-risk population (>36 million pregnancies) with over 12 million infected in sub-Saharan Africa annually^2^. Vaccines to protect pregnant women from malaria aim primarily to elicit antibodies to the *P. falciparum* virulence factor VAR2CSA that is uniquely expressed during infection in pregnancy^3^. VAR2CSA is expressed on the surface of infected erythrocytes and binds to chondroitin sulfate A (CSA)-specific proteoglycans on the placenta syncytiotrophoblast^4^, disrupting the exchange of oxygen and nutrients from the maternal blood to the fetus^5^. Placental malaria is associated with poor maternal and birth outcomes, including severe maternal anemia, low birthweight infants, pre-term birth and fetal growth restriction^6^, underscoring the importance of developing vaccines specific to this population.

Vaccines based on recombinant fragments of VAR2CSA showed promise in pre-clinical studies but failed to elicit broadly inhibitory antibodies in Phase I trials^7,8^. A key bottleneck for these vaccines is the extensive genetic diversity of the VAR2CSA alleles; thus, efforts are underway to identify conserved epitopes for next-generation vaccines^7,8^. One approach, common with genetically diverse pathogens like influenza and HIV, is to study the epitopes recognized by antibodies acquired naturally during infection^9^. Typically, VAR2CSA antibodies are acquired in a parity-dependent manner and are associated with protection from placental malaria^10^. However, antibodies to VAR2CSA were also reported in certain non-pregnant populations^11^. In Colombia and Brazil, men and children had antibodies to VAR2CSA at similar levels to pregnant women of all gravidities^12,13^. Based on the local epidemiology of malaria, we proposed that exposure to the *Plasmodium vivax* species, and specifically to the *P. vivax* Duffy binding protein (region 2; DBPII), can elicit antibodies that cross-react with VAR2CSA^11,14^. In support of this hypothesis, a mouse monoclonal antibody raised against DBPII, called 3D10, cross-reacts with VAR2CSA and moderately inhibits the adhesion to CSA of infected erythrocytes expressing different alleles of VAR2CSA^14,15^. These findings suggest a conserved epitope may be shared between these evolutionarily distinct proteins, which could be exploited to develop a vaccine against VAR2CSA^14,16^. A unifying trait between DBPII and VAR2CSA is the presence of Duffy binding like (DBL) domains, which are common to several malaria virulence factors used for invasion or sequestration.

Various studies describe the epitope of 3D10 in the DBPII region of PvDBP based on mutational analysis, phage display libraries, mimotopes, peptide arrays, and alanine scanning^17–19^. A discrete binding region was identified within subdomain 1 (SD1) that includes the amino acids NxxRKR and/or YK(R/Y/E). Yet a search for a similar motif in VAR2CSA failed to identify a homologous epitope, highlighting a common shortfall in mapping discrete epitopes recognized by cross-reactive antibodies^20^. Our screening of VAR2CSA by peptide array with 3D10 provided no clarity with regards to a conserved epitope between VAR2CSA and DBPII^21^. The array data revealed one major binding site along with several other clusters of highly reactive peptides within the VAR2CSA DBL domains but none of the peptides contained the consensus 3D10 epitope from DBPII^17,18^.

To resolve the ambiguity of the peptide array data and characterize our cross-reactive antibody, we employed machine learning to analyse the conserved features of 3D10 epitopes within two diverse alleles of VAR2CSA. We developed a regression machine learning framework to analyse 430 features from a dataset of over 4300 peptides in 4 arrays derived from DBPII, two alleles of VAR2CSA, and PvEBP2 (3D10 non-reactive). We used feature selection to extract physicochemical characteristics that describe 3D10 binding and applied those features to generate a panel of mutated peptides to test more complex patterns of amino acids. Our analyses revealed a broader set of criteria for 3D10 epitopes in VAR2CSA that are also present, as linear and conformational epitopes, in other DBL proteins from *Plasmodium*. We propose our framework as a method to generate a physicochemically-based binding profile of cross-reactive antibody binding^20^.

## 3. Results

### Machine learning and feature selection

**Figure 1.**
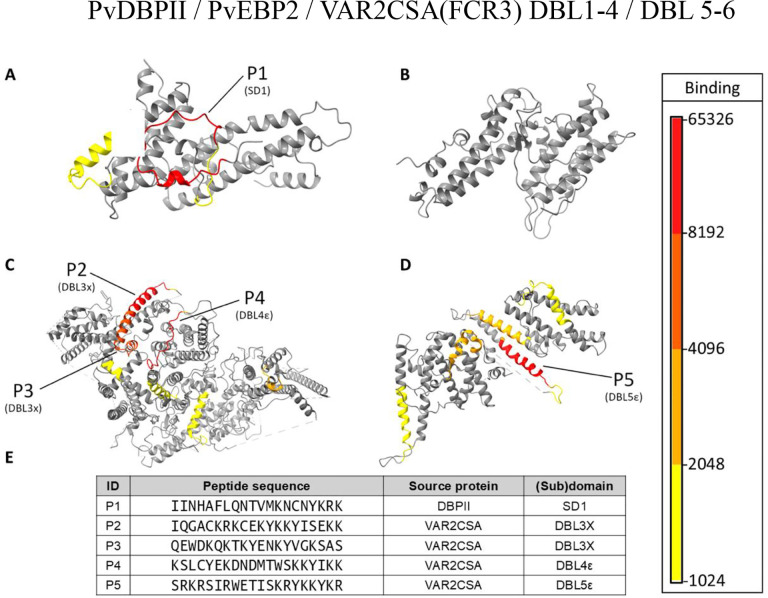
3D10 peptide array data mapped on their respective 3D protein structures. Structures generated by AlphaFold 3 (A-B) are accessible in a GitHub repository described in our data availability section. VAR2CSA (FCR3) structures were resolved by cryo-EM and are accessible at PDB ID 7JGE (C) and 7JGF (D)^23^. Raw median values are represented according to the colour scale and mapped to DBPII (A), PvEBP2 (B), VAR2CSA (FCR3) core region (DBL 1 to 4) (C), and VAR2CSA (FCR3) flexible arm region (DBL 5 and 6) (D). One peptide within SD1 and four peptides recognized most strongly by 3D10 in VAR2CSA were selected for further analysis (E). Figure 1 - PvDBPII / PvEBP2 / VAR2CSA(FCR3) DBL1–4 / DBL 5–6

The dataset used to develop the machine learning algorithm consists of the reactivity values (expressed as arbitrary units) of 3D10 to each peptide in the arrays derived from PvDBP, PvEBP2, and 2 alleles of VAR2CSA. We then mapped the peptide array data to 3D structures of DBPII, PvEBP2, and VAR2CSA (FCR3) to visualize 3D10 reactivity to these different proteins (Fig 1 A–D). As expected, 3D10 strongly recognized an epitope in SD1 of DBPII (Fig 1A). There were no epitopes in PvEBP2 (Fig 1B) consistent with previous findings that 3D10 does not recognize this recombinant protein by ELISA^16,22^. In VAR2CSA, 3D10 recognized several epitopes within DBL3 and 4 (Fig 1C) and DBL5 (Fig 1D). Based on the intensity of 3D10 binding, we identified classified peptides as 3D10 reactive and 3D10 non-reactive and identified the five most reactive peptides (**Fig S1**; Fig 1 E). Many of the strongest binding peptides contain a significant enrichment of lysine residues and tyrosines adjacent to lysines. However, these traits alone are insufficient to define reactive vs non-reactive peptides given that non-reactive peptides were also rich in lysines and tyrosines next to lysines was commonly observed in the dataset (**Fig S2**).

Next, we generated a panel of features (see methods) (**Table S1**) that we hypothesized were associated with 3D10 binding. These features focused on specific amino acids, plus properties such as charge, hydropathy and polarity. Structural features were included with the caveat that synthetic peptides can adopt heterogeneous structures *in vitro* that fail to replicate their structures within the native or recombinant protein^24,25^. The label (predicted variable) was the raw median 3D10 binding value of two replicates; this was selected to minimize the noise from non-reactive peptides. For feature selection, we applied the methods of feature elimination, feature utilization, and variance feature selection to our panel of 430 features, to select a small subset of features that correlated to 3D10 reactivity (see methods) (**Table S2**).

### Feature validation

As the features identified by our framework could be positively or negatively associated with 3D10 binding, the next step of validation was to discern which features define 3D10 epitopes. We analyzed the dataset using three methods: heatmaps of features, amino acid enrichment calculation, and analysis of amino acid distances within each peptide (see methods).

**Figure 2.**
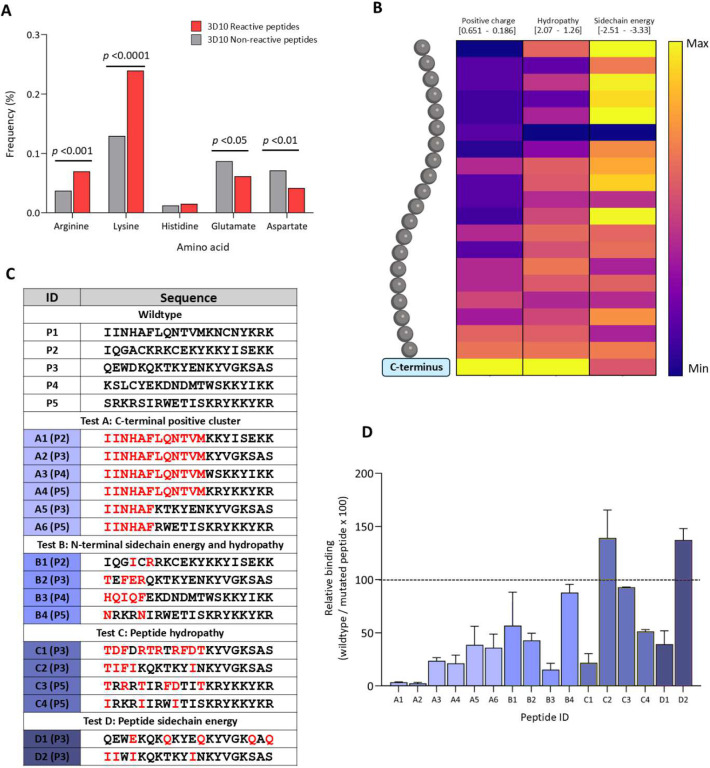
Binding-correlated clusters of C-terminal lysines and arginines are insufficient for 3D10 reactivity. (A) Frequency of charged residues in 3D10 reactive and non-reactive peptides was compared. Significance was calculated by two-way ANOVA assuming a Gaussian distribution. (B) Heatmap of positive charge, sidechain energy, and hydropathy in 3D10 reactive peptides. Each cell indicates a residue position from the N-terminus at the top to the C-terminus at the bottom. The colour scale for each table ranges from the maximum to minimum for each feature as indicated above each column. (C) Table of peptide sequences with mutated residues coloured red. Peptides are grouped by different tests. (D) Mutated peptide reactivity relative to wildtype. Bars are coloured relative to their test group.

The first set of analyses focused on positive charge, hydropathy, and sidechain energy (Fig 2). In 3D10 reactive peptides, we observed that lysine and arginine residues were significantly enriched (Fig 2 A). There was also a tendency for positive residues to cluster at the C-terminus and for non-polar residues to cluster at the N-terminus of 3D10 reactive peptides (Fig 2 B)^26,27^. We hypothesized that N-terminal modifications of VAR2CSA peptides that preserved the C-terminal positive clusters observed in P2 to P5 may increase reactivity as they will be more like P1, the minimum binding peptide from DBPII. To test this hypothesis, we designed subsets of mutated peptides for four different tests (Fig 2 C). For each subset, we applied different approaches to adjust the relative hydropathy and sidechain energy of P2 to P5 to be closer to P1. In subset A, we tested if hybrid peptides that combined the N-terminal residues of P1 with the C-terminal positive clusters of P2-P5 were sufficient or enhanced binding to 3D10; this would indicate N-terminal sidechain energy and hydropathy contribute to the differences in reactivity between peptides. In tests B, C, and D, we mutated residues in peptides P2 to P5 to set the overall peptide sidechain energy and/or hydropathy to be similar to P1. We used either chemically related amino acids, or a minimal number of amino acids while avoiding (where possible) changes to positively charged residues (Fig 2 C). We tested mutated peptides by indirect ELISA and binding was compared to the non-mutated ‘wildtype’ peptides (Fig 2 D). We observed that the N-terminal modifications in P2 to P5 decreased binding in all subsets except when modifications decreased the total number of negative residues. Additionally, the clusters of positive residues at the affixed C-terminus, despite being strongly associated with 3D10 reactive peptides, were not sufficient to explain 3D10 reactivity to peptides P2 to P5. Moreover, sidechain energy and hydropathy patterns at the C-terminus that correlate to 3D10 reactive peptides involve a more complex signature than what is observed in our heatmaps (Fig 2 B)^26,27^.

**Figure 3.**
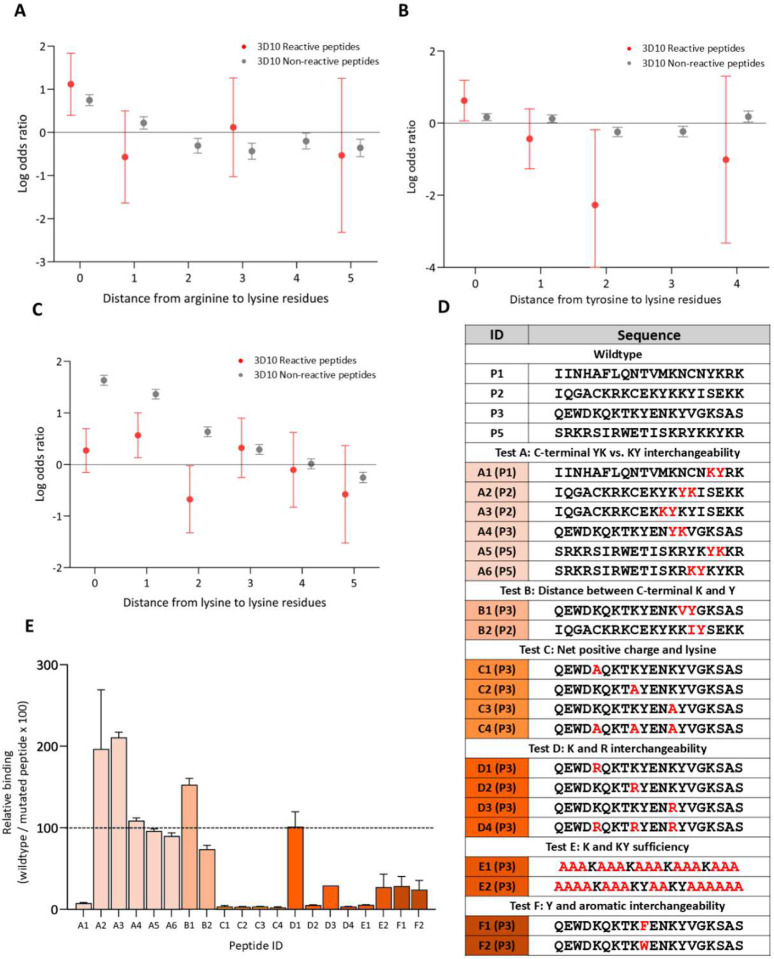
Lysine adjacent to tyrosine and specific positive residue patterns result in strong 3D10 reactivity. (A-B, C) Log odds ratio of distances between arginine to lysine, tyrosine to lysine, or lysine to lysine residues comparing observed proportion to expected value in 3D10 reactive and non-reactive peptides. Missing data points indicate a distance value occurred zero times and is significantly less common than expected (see methods) (D) Table of peptide sequences with mutated residues coloured red. Peptides are grouped by different tests. (E) Mutated peptide reactivity relative to wildtype. Bars are coloured relative to their test group.

Our analysis suggested that clusters of positive charge at the C-terminus were insufficient for 3D10 reactivity, and reactivity was affected by N-terminal mutations modifying net-charge (Fig 2 C–D). To expand on these observations, we increased the depth of positive charge analysis and characterized sequence patterns relative to enriched positive residues (Fig 3 A). Consistent with the heatmaps of 3D10 reactive peptides, there was an increased likelihood of lysine residues positioned adjacent to arginine residues (Fig 3 A; Fig 2 B). Tyrosine residues were significantly more likely to occur adjacent to lysine residues and significantly less likely to occur distal from lysine residues; this corresponds with the selection of the ‘KY count’ feature (Fig 3 B). Further, lysine residues had a higher propensity to occur one amino acid away from other lysine residues (KxK) in 3D10 reactive peptides (Fig 3 D). ‘YK’ was not selected as a feature despite being present in P1. To determine if the absence of ‘YK’ in selected features was a function of the dataset, we swapped the position of lysine and tyrosine residues to create the opposite feature (Fig 3 D). We also decreased the ‘KY’ count by increasing the distance between lysine and arginine (test B). Again, we focused on C-terminal ‘K’ and ‘Y’ residues as our top-binding peptides consistently contained C-terminal ‘KY’ motifs. To test N-terminal positive residues, we focused on P3 as it contained positive residues with consistent spacing allowing us to explore the interchangeability of lysine and arginine residues and the requirement for specific lysine residues (tests C, D, and E). Finally, we tested whether tyrosine could be interchanged with other aromatic residues (test F) (Fig 3 C, E). Consistently, we observed that despite the chemical similarities of arginine and lysine or phenylalanine and tyrosine, it was essential that lysine was adjacent to tyrosine for high 3D10 reactivity. This highlights that 3D10 binding requires the specific amino acid motif: ‘KY’/‘YK’.

**Figure 4.**
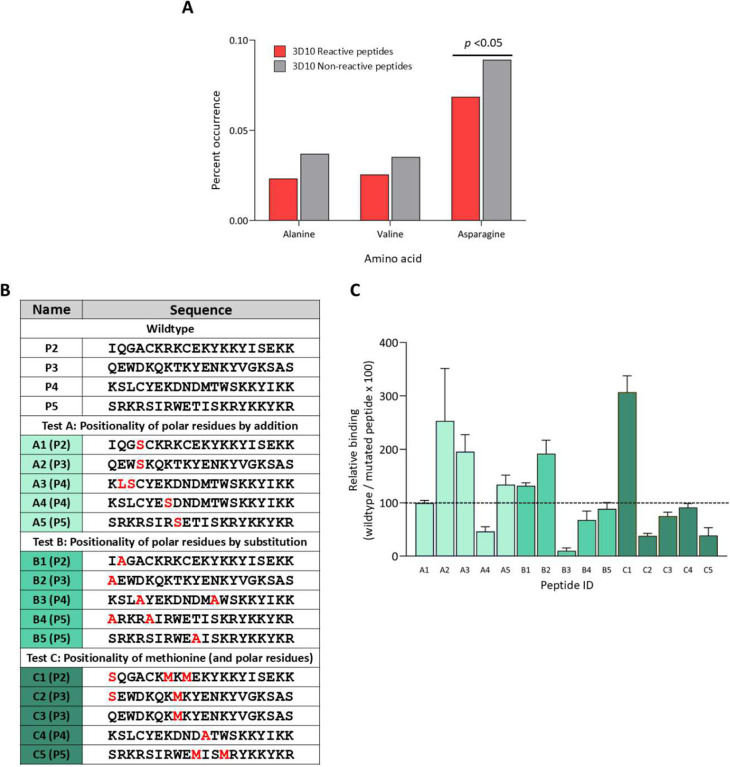
Polar residues do not consistently predict binding of 3D10. (A) The frequency of residues in 3D10 reactive and non-reactive peptides was compared. Significance was analysed by two-way ANOVA. (B) Table of peptide sequences with mutated residues coloured red. Peptides are grouped by different tests. (C) Mutated peptide reactivity relative to wildtype. Bars are coloured relative to their test group.

For several of our selected features, the statistical analyses did not uncover significant differences between 3D10 reactive and non-reactive peptides (**Table S2**). However, their selection by the decision trees suggested they could contribute to 3D10 reactivity. These include non-polar residues, specifically VM, VN, VT, VQ, MT, alanine, and valine counts. Also, there was a tendency for alanine and asparagine to occur less frequently in 3D10 reactive peptides (Fig 4 A). It is unclear if the reduced frequency of these residues is due to the proportional increase in positive residues like lysine and arginine (Fig 2 A). Additionally, we were unable to determine a distinct pattern relative to hydropathy by broadly adjusting the hydropathy of 3D10 reactive peptides (Fig 2 B, D). This may highlight a more complex relationship that varies from peptide to peptide. To address this, we mutated minimal polar and non-polar residues into various positions to determine their importance. For tests A, B, and C, we used serine, methionine, and alanine: serine was present in all top-binding peptides, methionine was present in the frequently observed ‘VM’ feature and alanine was used to mutate polar residues (Fig 4 B). The results of this testing failed to establish a specific pattern of polar residues relative to other aspects of the 3D10 epitope (Fig 4 C). These results suggest that polarity is not a sufficient criterion to distinguish 3D10 reactive peptides from 3D10 non-reactive peptides.

By integrating the results from all mutant peptide sets, we propose the following six criteria for highest 3D10 reactivity: 1) a lysine adjacent to a tyrosine; 2) a lysine one residue away from another lysine; 3) clusters of lysines and arginines; 4) a polar residue proximal to the criteria 1/2/3; 5) minimal negative residues within or between the above criteria; and 6) criteria 1–4 are proximal to one another. The first criterion, a tyrosine adjacent to lysine, is present in 40 of 43 3D10 reactive peptides and only one amino acid away in the remaining peptides. We found that this criterion is not restricted to the C-terminus (Fig 3 E). We also observed that arginine cannot replace lysine, nor can other aromatics replace tyrosine (Fig 3 E). We observed a significantly higher rate of lysine positioned one amino acid away from another lysine (Fig 3 D). Particularly, we observed that binding was diminished or enhanced with the deletion or addition of the ‘KxK’ motif, respectively (Fig 2 C–D; Fig 3 D–E). We observed that arginine and lysine residues were positioned proximal to one another, positive charge was strongly correlated to binding, and these residues clustered at the C-terminus of reactive peptides (**Table S2**; Fig 2 A–B; Fig 3 A–C). Our data indicated that this clustering is not sufficient for 3D10 reactivity but modifications in this region resulted in a major loss of 3D10 reactivity (Fig 2 D; Fig 3 E). We observed that the addition or removal of certain polar residues had significant effects on reactivity, but we were unable to discern a specific positionality relative to other criteria necessary for reactivity (Fig 2 D; Fig 4 C). The addition of negative residues consistently decreased binding while the mutation of negative residues increased binding. Finally, because 3D10 strongly reacts to linear peptides, like P1, we believe that ideally, these criteria must be proximal to one another; in P1, all criteria are satisfied in the minimum possible 6 amino acids^28^.

### 3D10 binding criteria application and testing

To validate these criteria within the context of a protein with three-dimensional structure, we constrained the distance between criteria to a field of ~1000 Å^2^ and restricted our analysis to epitopes on the protein surface^29–31^. Moreover, we defined strict standards to translate criteria for 3D structure mapping. We defined clusters of arginines and lysines as 3 of either residue present within a 6 amino acid span. Criterion 2 was defined as ‘KY’ or ‘YK’ occurrences in protein sequence, and criterion 3 was defined as any occurrence of ‘KxK’ excluding ‘KDK’ or ‘KEK’. ‘Proximal’ was defined as continuous on the surface structure without negative residues between criteria 1, 2, or 3. This standard eliminated ‘KEK’ and ‘KDK’ from consideration. Criterion 4 was interpreted to be the occurrence of a polar residue continuous with any of criteria 1, 2, or 3.

**Figure 5.**
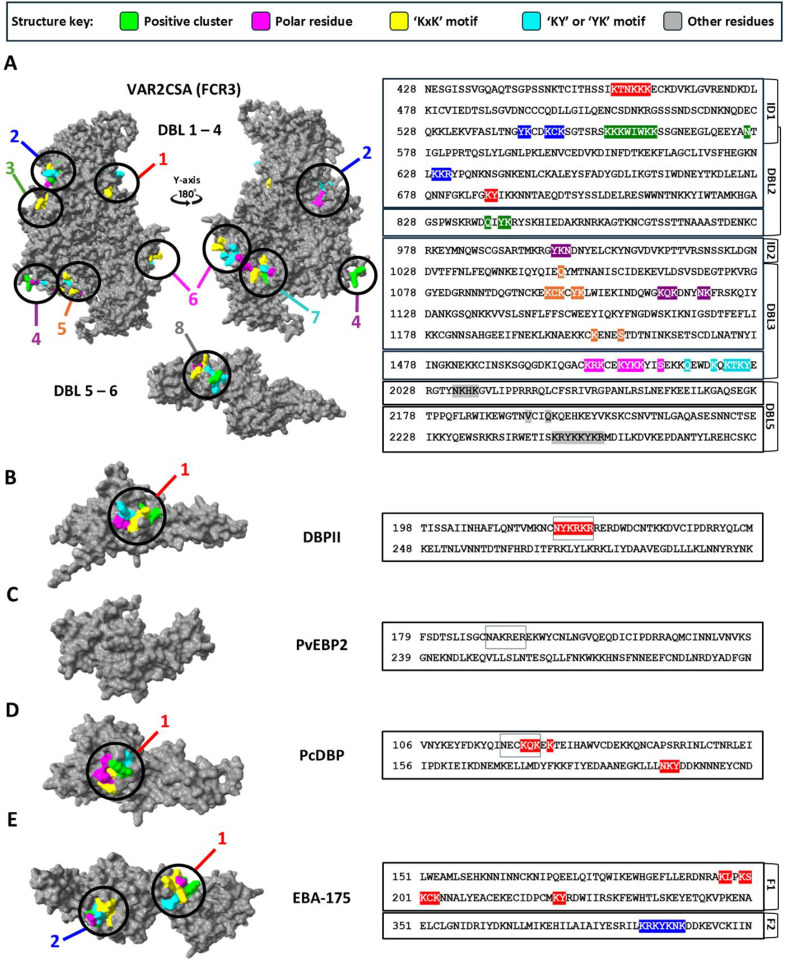
Mapping of proposed 3D10 binding criteria onto 3D structures of *Plasmodium* proteins. Structures generated by AlphaFold 3 (A-D) are accessible in a GitHub repository described in our data availability section. EBA-175 structure was resolved by crystallography and is accessible at PDB ID 1ZRL (E)^35^. (A-E) Surface-exposed regions containing a polar residue (magenta), a ‘KxK’ motif (yellow), a ‘KY’ / ‘YK’ motif (cyan), and a positive cluster region (green) that are uninterrupted by other residues or negative residues (grey and red) within a 28-Angstrom diameter was defined as satisfying the criteria. Criteria mapped onto the structure of VAR2CSA (FCR3) (A), DBPII (B), PvEBP2 (C), PcDBP (D), EBA-175 (E). Regions that satisfy the criteria are highlighted and numbered relative to their order in the protein sequence and assigned a specific colour. Boxes around specific amino acids indicate homologous regions between DBPII (B), PvEBP2 (C), and PcDBP (D).

We classified protein segments that satisfied all six criteria as ‘signature regions’. In our analysis of VAR2CSA (FCR3 allele), we observed 8 signature regions across the protein (Fig 5 A). We identified 7 signatures in the VAR2CSA core region DBL 1 to 4. One signature includes the sequences from P2 and P3. P4 was not associated with any of the signatures because it was not surface accessible and does not satisfy criterion 2^21^. One signature was identified in DBL5 within the VAR2CSA arm region and mapped to P5. Surprisingly, several of the signature regions are discontinuous and predicted to be conformational epitopes (Fig 5 A). To further validate both the specificity of this approach and the breadth of cross-reactivity of 3D10, we mapped the 3D10 binding criteria onto other DBL proteins from *Plasmodium* (Fig 5 B–E)^32^. We used the DBPII region of PvDBP as a reference, and as expected, the criteria mapped to the known epitope in SD1 (Fig 5 B)^18^. Consistent with our array data and experimental data, PvEBP2 was not predicted to have any signature regions despite containing a DBL domain (Fig 1B; Fig 5 C)^16,22^. We extended our analysis to PcDBP, an orthologous protein from *Plasmodium chabaudi* that is closely related to DBPII and PvEBP2. We identified a single conformational signature corresponding to the same region in DBPII (Fig 5 D). EBA-175 from *Plasmodium falciparum* also shares significant genetic similarity to the DBL domain of DBPII and does contain 2 signature regions: one predominantly linear and one conformational (Fig 5 E)^22,33,34^.

**Figure 6.**
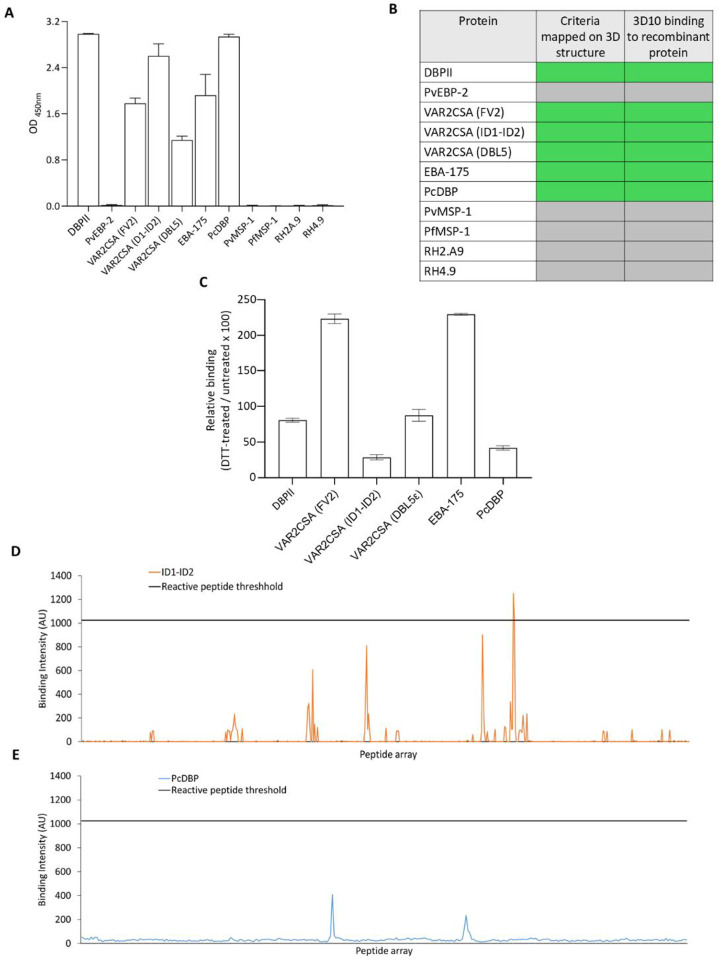
Validation of epitopes predicted by 3D10 binding criteria. (A) ELISA reactivity of 3D10 against a panel of recombinant proteins. (B) Comparison of experimental and predicted binding of 3D10 to various *Plasmodium* antigens. 3D10 binding to peptide arrays was defined by any peptide in the array that could be classified as 3D10 reactive, as characterized in **Figure S1**. Green shading indicates binding, or satisfaction of criteria and gray indicates no binding or failure to satisfy criteria. (C) Reactivity of 3D10 to DBL proteins treated with DTT to disrupt disulfide bonds. (D-E) Peptide array reactivity for peptides spanning the ID1-ID2 region of VAR2CSA (FCR3) (D) and PcDBP (E).

To validate the predicted binding signatures and test the specificity of these criteria, we tested 3D10 against a panel of recombinant proteins by ELISA (Fig 6 A). Except for PvEBP2, the presence of Duffy binding-like (DBL) domains consistently corresponded to 3D10 binding; DBL domains are present in 3D10 reactive proteins (DBPII, VAR2CSA (FV2), VAR2CSA (ID1-ID2), VAR2CSA (DBL5ε), EBA-175, and PcDBP). None of the other *Plasmodium* proteins were recognized by 3D10. Importantly, in all cases, 3D10 binding *in vitro* validated the predictions *in silico* (Fig 6 B). An exciting outcome of the algorithm is that many of the epitopes within these proteins were predicted to be conformational, specifically within ID1-ID2, DBL50ε and PcDBP, and one of two predicted epitopes in EBA-175. We tested whether disruption of the disulfide bonds in these DBL domains would reduce binding of 3D10 (Fig 6 C). As expected, there was no change in reactivity to DBPII which contains a linear epitope. There was also no change in reactivity to DBL5ε, whereas reactivity increased to the full-length VAR2CSA and EBA-175, suggesting DTT treatment may reveal linear epitopes that are not surface-exposed. Of specific interest, DTT reduced binding of 3D10 to ID1-ID2 and PcDBP, consistent with these epitopes being conformational. To validate further, we examined the array data for the ID1-ID2 region and noted only one weakly reactive linear peptide was recognized above our signal-to-noise threshold (Fig 6 D). We also tested 3D10 binding to an array of linear peptides spanning the DBL domain of PcDBP and no linear peptides were recognized (Fig 6 E).

## 4. Discussion

**Fig 7.**
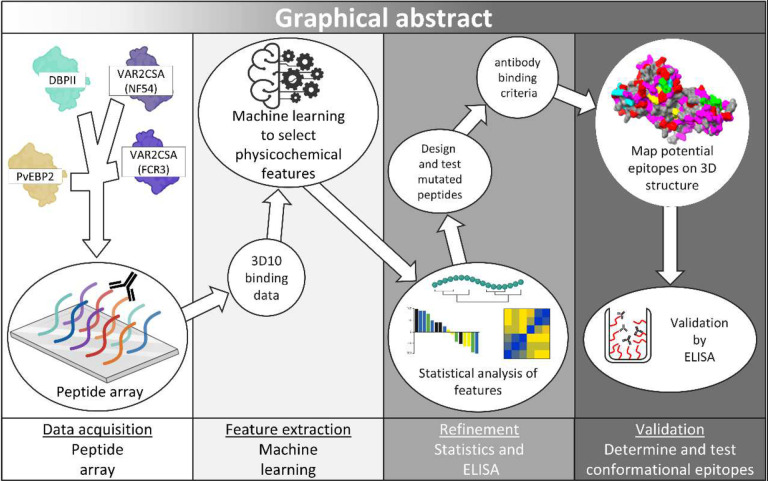
Machine learning peptide array analysis framework. This flowchart describes the process to extract 3D10 binding properties from peptide array analysis. Divisions indicate different stages of experimentation. Assets partially generated with Biorender.com.

We propose a framework for integrating machine learning to enhance epitope mapping with peptide arrays (Fig 7). In this pipeline, peptide array data were combined with feature selection to extract features associated with antibody binding (positively or negatively); statistical analysis and empirical testing down-selected specific binding criteria, which were then mapped onto 3D protein structures to predict linear and conformational epitopes. We identified six criteria for 3D10 binding which mapped to predicted epitopes only in those proteins that were recognized by 3D10 experimentally. These criteria are consistent with the 3D10 epitope in DBPII requiring the amino acids ‘NxxRKR’ and/or ‘YK(R/Y/E)’ determined by mutational analysis, arrays, and mimotopes^17,18^. Importantly, our criteria identified additional physicochemical features that broaden the epitope beyond this simple motif, which in turn, explains the cross-reactivity of 3D10 with VAR2CSA.

When we mapped the criteria to the VAR2CSA structure, we identified 8 predicted epitopes. Two of these correspond to a linear sequence in DBL3X that matches two peptides (P2 and P3) recognized most strongly by 3D10 in the array. Surprisingly, the other 6 epitopes are predicted to be conformational. These findings suggest that the framework can reveal conformational epitopes based on linear array data. This was evident with the related DBL protein, PcDBP, which was recognized by 3D10 by ELISA and western blot (data not shown) but failed to bind to any linear peptides in an array. Since DBL domains are structurally stabilized by disulphide bonds^36,37^, we used DTT to disrupt the sole conformational epitope and indeed, recognition by 3D10 was reduced. Other approaches such as HDX, cryo-EM and site-directed mutagenesis could further validate each of the predicted 3D10 epitopes in these various DBL proteins. While DTT reduced the binding of 3D10 to PcDBP and ID1-ID2, we noted that binding to DBL5ε was not affected and for EBA-175 and the full-length VAR2CSA, binding increased. We propose that DTT treatment can expose other linear peptides that are not surface-exposed. In VAR2CSA, the peptides P4 and P5 were among the most reactive in the array data yet did not meet the criteria from the algorithm because they did not map to the protein surface. When the disulfide bonds were disrupted, these linear epitopes may become accessible for binding to the mAb.

One concern is that 3D10 is a polyreactive antibody that binds promiscuously to all DBL proteins. Analysis of DBL domains from *Plasmodium* proteins suggests a high likelihood for specific signatures such as ‘KxK’ and positive clusters despite significant sequence differences and genetic diversity^38,39^. However, our data with PvEBP2 (which shares 36% sequence identity and 52% sequence similarity with DBPII (**Fig S3**)) support the specificity and accuracy of our 3D10 binding criteria. 3D10 does not bind to the PvEBP2 recombinant protein nor to any of its peptides within our array. When we applied the six criteria for binding to the 3D structure of PvEBP2, no epitopes were predicted. The implication is that the criteria generated by our framework and the requirement for those criteria to be satisfied within a defined physical space on the surface of the protein were specific enough to differentiate reactive and non-reactive recombinant DBL domains. If criteria are not sufficiently restrictive, the framework will be subject to a high rate of false positive signature prediction. For example, our criterion 4 concerning polar residues did not eliminate any signatures for our proteins, and this criterion’s low specificity was not meaningful for predicting 3D10 binding. If we established a definite pattern for the position of polar residues for 3D10 reactivity, we might eliminate one or several potential signatures in VAR2CSA.

The generation of restrictive criteria largely depends on the design and size of the peptide arrays used to train a machine learning model. Although we used arrays specific for target antigens, non-natural randomized peptide arrays could be screened with an antibody of interest to extract features associated with binding or performed in addition to target antigens. The ideal panel would have high variance with respect to physicochemical features evaluated by machine learning and several peptides with varying levels of reactivity. Additionally, conformationally constrained peptide arrays could be incorporated into testing and feature space to enable the consideration of certain structural elements during criteria development^40–42^.

Based on our findings, we propose that 3D10 is an example of a cross-reactive monoclonal antibody, a type of antibody specific for a few antigens related via their DBL domains^43–46^. Recent work has labelled similar antibodies as “super antibodies” or “promiscuous” and note that these antibodies form a population distinct from typical highly specific antibodies^9,20,47,48^. This type of antibody is associated with antigenically variable pathogens; much like DBL represents an antigenically variable domain present in many malaria proteins important for pathogenesis^49–52^. Despite consistent structural elements (such as interdomain and intradomain disulphide bonds), DBL domains of pathogenic *Plasmodium* proteins are genetically diverse^36,52,53^. A review by Walker et al. 2018 highlighted the association between antigenic variability and the generation of broadly neutralizing antibody responses^9^. For example, HIV and influenza often elicit antibodies with cross-reactivity to related antigens, supporting development of ‘universal’ vaccines for these immune-evasive pathogens^51,54–57^. These antibodies make up a fraction of the total pool of antibodies but have become a focus in the development of vaccines against immune evading pathogens like HIV^58^. These efforts are tempered by concerns that these antibodies may be difficult to elicit consistently, and not all are broadly neutralizing^59^. Our framework and approach may serve as an effective platform for the comparison of these antibodies to one another and to identify common elements between them. As more broadly neutralizing antibodies are discovered against HIV, our framework can be used to determine physicochemical patterns that are associated with high breadth and potency of neutralization. The conserved structural and physicochemical properties of multiple binding regions for cross-reactive monoclonal antibodies can be exploited to effectively target antigenically variable pathogens with vaccines.

If applied to polyclonal sera, this framework can also be used to extract features associated with immunodominance. For specific pathogens such as influenza, the criteria could define specific immunodominant features within the HA head region (for example) that elicit only strain-specific antibodies, resulting in low vaccine efficacy^60^. Targeted epitope masking strategies could be applied to reduce the immunogenicity of these sites and stimulate responses to subdominant, protective epitopes.

The key utility in this framework is a new interpretation of peptide array data. Specifically, it can analyze peptide array data as a physicochemical characterization of the interface between an antibody and thousands of different antigens rather than a tool to map discrete epitopes by residue. Machine learning enables the simultaneous analysis of these antibody-antigen interfaces to reveal complex features of epitopes that extend beyond identifying discrete amino acid motifs. This approach to cross-reactive antibody characterization presents a marked shift from existing methods to better understand their target epitopes and can be applied to both linear and conformational epitopes.

## 5. Methods

### 5.1 Peptide array and data processing

Synthetic peptide arrays were synthesized by PepperPrint^™^ (Germany) using the PEPperCHIP protocol to develop overlapping arrays: VAR2CSA (FCR3) (GenBank: AAQ73926.1, accession: AY372123.1, residues 1–2659), VAR2CSA (NF54) (GenBank: EWC87419.1, accession: KE123842.1, residues 1–2652), DBPII, PcDBP, and PvEBP2. Peptides were 20 amino acids in length and there was a 19 amino acid overlap between peptides in all arrays except VAR2CSA (NF54) for which there was an 18 amino acid overlap.

For each data point, we used the average of the median foreground intensities for each spot (arbitrary units). For each peptide, binding was measured in duplicate (V1 and V2), and the average raw median intensity was used except for when the difference between the lesser of the two values was less than or equal to 40% the larger of the two values.


MIN(V1,V2)<=MAX(V1,V2)*0.4

Additionally, some peptides occurred in both VAR2CSA (FCR3) and VAR2CSA (NF54); in this case, the highest of the two averaged values were taken. This resulted in a total of 4,378 data points being left; 215 duplicate data points were identified and removed. We employed a test-train split of 1/3 to 2/3 to randomly distribute the data. That is, 2/3 of the datapoints and randomly selected to be involved in training and cross-validation phases of algorithm development and 1/3 of datapoints are selected to test the algorithm.

### 5.2 Features

Features employed in machine learning can be placed into six categories: single amino acid counts, dipeptide counts, secondary structure, physicochemical values, charge, and disulphide bond potential (**Table S1**). These values are either whole number, continuous, or binary values. Single amino acid counts are the total counts of each of the 20 different amino acids occurring in protein sequences. The secondary structure employs S4PRED to predict the secondary structure of the recombinant protein sequences, including the elongating “GSGSGSG” N-terminal and C-terminal linkers, and values were taken as the average of residue values for respective peptide sequence segments^61^. There were three values for secondary structure representing the odds of forming either an alpha-helix, beta-sheet, and a flexible coil. Physicochemical values are based on sums of values for amino acids from 3 different physicochemical scales: sidechain energy, hydropathy, and polarity^26,27,62^. Charge features were sums of positively charged residues (lysine, arginine, and histidine), negatively charged residues (glutamate and aspartate), and net charge. Disulphide bond potential is a binary feature which is set to ‘1’ if there are two cysteine residues in a peptide with two or more amino acids in between. Dipeptide counts, like single amino acid counts, is a count of total occurrences of each combination of 2 consecutive amino acids; 400 for each combination of 20 amino acids. This feature is adjusted by the hyperparameter ‘peptide trim.’ ‘Peptide trim’ for dipeptide counts defines the number of amino acids to skip before counting dipeptide features. This is employed because of the concern that the C-terminal of the peptides may be less accessible to 3D10 as this is the end attached to the glass slide. We chose not to employ this hyperparameter for other features as dipeptide count is intended to identify patterns of specific amino acids, whereas single amino acid counts, charge, etc., inform the physical properties of the whole peptide, which may affect its flexibility or be used in conjunction with other features.

### 5.3 Feature selection

We employed three methods for feature selection: variance-based feature selection, feature utilization, and recursive feature elimination. Using an ensemble of features accounts for the potential of each method to be biased for missing predictive features and selecting nonpredictive features. Both variance-based feature selection and recursive feature elimination are provided by scikit-learn^63^. We created the feature selection method feature utilization for our project.

Variance-based feature selection is an unsupervised method, meaning it is agnostic to 3D10 reactivity. The goal of this method is to establish which features have high variance and, therefore, are less likely to be selected due to overfitting. Because it is not dependent on 3D10 reactivity, features selected by this method are not sufficient to qualify them for analysis. We used a threshold (p) of 1.


Var|X|=p(1-p)

Feature utilization is not available in Scikit-learn. Because of the random nature of our train-test-splitting, this method involves quantifying feature usage frequency from the lowest squared error trees of 100 tournaments of 100 randomly generated trees. If any feature is present in a tournament-winning tree, it is defined as being ‘selected.’

Recursive feature elimination recursively eliminates features to a given value until a cohort of predictive features is left. Starting with the whole feature set (n features) will eliminate a poorly correlative feature to 3D10 reactivity and yield ‘n-1’ features. It repeats this until it reaches our defined values of 5, 10, or 15 features. Fifteen features represent the maximum number of features that can be applied by a tree with depth 4.

### 5.4 Statistical methods and feature analysis

We used 3 methods to analyze features in addition to their selection: heatmapping, enrichment, and sequence patterns. For enrichment analysis, we compared 3D10 reactive peptides to 3D10 non-reactive peptides (**Fig S1**).

For heat mapping, we determined average feature values for each of the 20 positions amino acid positions that span the length of the peptides. As the peptide array contained significantly overlapping peptides, the average value for each position in the non-reactive population was almost identical to that of the average for the whole sequence. This meant that the comparison between non-reactive peptides and reactive peptides was not meaningful and may diminish trends observed in 3D10 reactive peptides if they were reflected in the whole sequence. For example, 3D10 reactive peptides were more likely to contain positively charged residues than non-reactive peptides; the difference in their average positive charge for each residue position does not improve the characterization of positionality of positive residues. The data provided by the difference between 3D10 reactive and non-reactive peptides would be better characterized by enrichment analysis.

Amino acid enrichment compared frequencies of amino acid occurrence in 3D10 reactive peptides and 3D10 non-reactive peptides. Statistical significance was determined by 2-way ANOVA selecting for relevant comparisons and correcting for number of comparisons (only comparing values for each amino acid between reactive and non-reactive peptide groups). For example, to compare three different amino acid occurrences in either group, we corrected for three comparisons.

The method for determining amino acid patterns is similar to the method for sequence analysis known as TMSTAT^64^. Like TMSTAT, we do not have to assume that the distribution of amino acids across sequences is not anti-segregated or co-segregated relative to the features we are seeking to analyze. Assuming non-anti-segregated and non-co-segregated distribution assumes that amino acid distributions are homogeneous across all populations. We do not need to perform this assumption because our statistical comparisons are between observed odds and the expected odds. The expected odds, in this case, would be the likelihood of a homogeneously distributed population. For two types of amino acids, ‘A’ and ‘B’, we calculated the expected probability of a distance between an ‘A’ amino acid to the closest ‘B’ amino acid in 3D10 reactive or 3D10 non-reactive peptides, $P_{\text{Exp}}$. This value depended on the average number of ‘B’ amino acids in the respective population, $\overset{‾}{B}$. Expected odds for any distance calculated by bootstrapping. We simulated all possible distributions of 1 to 5 ‘B’ amino acids across the peptide. We determined the shortest distance between an ‘A’ amino acid to the closest ‘B’ amino acid to develop a reference table (**Table S3**). For each possible distance, $D^{X}$, we obtained a probability as a function of $\overset{‾}{B}$ where X denotes the discrete minimum distance between ‘A’ and the closest ‘B’ amino acid; X∈{1,2,3,…,19}. This probability function was calculated by performing second-order polynomial regression on the bootstrapped odds.


$$P_{\text{Exp}}=f\left(D^{x}|\overset{‾}{B}\right)$$

This resulted in 19 equations for each possible value of X and enabled the prediction of expected minimum distances between ‘A’ and ‘B’ type amino acids for cases where $\overset{‾}{B}$ was not a whole number. The observed probabilities were a proportional count for the occurrence for each distance to occur in the population $P_{Obs}$. Log odds ratio was obtained by a ratio of observed distance proportions and expected distance probabilities.


$$\text{LN}\;\left(\frac{P_{\text{Obs}}}{P_{\text{Exp}}}\right)$$

We used 95% confidence intervals as a significance cut-off and calculated using previously published methods that assume a normal distribution^65^. With this cut-off, we could establish if the increased likelihood for a particular distance to occur between amino acids of type ‘A’ and ‘B’ was significantly more than expected. For situations where ‘A’ and ‘B’ were the same (like lysine-to-lysine distances), we employed a correction of $\bar{B_{\text{Cor}}}=\overset{‾}{B}-1$ to account for the fact that the reference lysine serving as ‘A’ will not be contributing to $\overset{‾}{B}$ relative to the expected probability of distances. We ignored cases in which only ‘A’ or ‘B’ were present as distances between them would be indeterminant. For a specific $D^{X}$, we excluded plotting data points for which the $P_{\text{Obs}}$ was 0 as this meant that the observed probability was indeterminately less common than $P_{\text{Exp}}$ as the log odds ratio is equal to –∞. For graphing, we plotted $D^{\left[0,5\right]}$ as the 95% confidence interval grows as $P_{\text{Exp}}\to 0$.

### 5.5 Indirect ELISA

We measured 3D10, a mouse IgG Mab, reactivity by an enzyme-linked immunosorbent assay (ELISA). 96-well Maxisorb plates (catalog no. 439454; Thermo Fisher Scientific) were coated with synthetic peptides at 10 μg/mL and recombinant proteins at either 1 μg/mL (VAR2CSA ID1-ID2) or 0.5 μg/mL (all others) at 50 μL diluted in 1 x PBS and incubated overnight at 4°C. We then washed wells once with 4% bovine serum albumin (BSA, catalogue no. A7906; Sigma-Aldrich) and incubated wells with 4% BSA for 1 h at 37°C. We washed wells 4 times with 1X PBST (0.1% Tween 20). Plates were incubated with 50 μL of primary antibody for 1 h at room temperature (RT) and washed four times with 1 x PBST. Plates were incubated with primary antibody for 1h at room temperature (RT) and washed four times with 1 x PBST. We then applied the secondary antibody treatments. Primary antibody treatments were either 3D10, Mouse IgG1 Isotype Control, catalogue no. MA5–14453 (Invitrogen, Waltham, MA)- Isotype Control, catalog no. MA5–14453, or 2% BSA. We repeated washing with 1 x PBST (0.1% Tween 20). We then incubated samples at RT for 1 h with 50 μL of HRP-conjugated secondary antibody (goat anti-mouse HRP, catalog no. 170–6516, Bio-Rad, Mississauga, Canada). After repeating washes, 50 μL of 3,3′,5,5′-Tetramethylbenzidine (TMB, catalog no. T0440; Sigma-Aldrich) was added and incubated for 30 min or when reactions approached saturation. This consideration was relevant for cases in which the optimized antibody concentration for a wildtype peptide (P1 to P5) resulted in saturation OD values for mutated peptides that significantly increased reactivity. We stopped reactions with 50 μL H_2_SO_4_ (0.5 N). All samples were tested in duplicate on the same plate and at least two plates were tested on different days. For ELISAs with protein segments treated with DTT, samples were incubated with DTT (10mM) at 56°C for 10 min and then added to the plate. The optical density (OD) values were corrected by subtracting the average of blank wells from the raw measurements and evaluating if IgG isotype control and secondary antibody control had no reactivity (OD<0.25).


$$\text{OD}={\text{OD}}_{\text{measured}}-{\text{OD}}_{\text{blank}}$$

To determine relative binding, OD values for mutated peptides were measured as a ratio to the wildtype peptide from which they are derived:

$$\text{Relative}\;\text{binding}=\left({\text{OD}}_{\text{mutated}}/{\text{OD}}_{\text{wildtype}}\right)\;*100$$

Several of the reagents crucial to my project were gifts or generously provided from various sources. The 3D10 antibody was generously provided by Dr. John Adams. EBA-175 was obtained through BEI Resources, NIAID, NIH: Plasmodium falciparum Erythrocyte Binding Antigen-175 RII-Non-Glycosylated Protein, Recombinant from Pichia pastoris, MRA-1162, contributed by Annie Mo. Our reagents, RH2.A9 and RH4.9-HIS TAG sequences, have been previously described^66–68^. Pv200MSP1_19_ was obtained through BEI Resources, NIAID, NIH: *Plasmodium vivax* yP30P2-Pv200 MSP1_19_ Protein, Recombinant from *Saccharomyces cerevisiae*, Strain 2905/6, MRA-60, contributed by David C. Kaslow. Whole VAR2CSA (FCR3) and VAR2CSA (FCR3) (ID1-ID2) were generously provided by Dr. Ali Salanti. Collaborators E. Medawar and A. Jin produced recombinant VAR2CSA (FCR3) (DBL5ε) protein.

### 5.6 Criteria mapping and structure prediction

To map criteria onto 3D structures, we first generated structures of relevant protein segments in AlphaFold 3^32^. For proteins that had high-resolution structures available, we used the experimentally determined structures. Only EBA-175 and PvMSP1 structures were sourced from previously resolved structures. We used AlphaFold 3 generated structures because of the unavailability of target protein structures, or, in the case of VAR2CSA, available protein structures were insufficient. More specifically, previously resolved structures for VAR2CSA (FCR3) and (NF54) had several surface-exposed segments excluded^23,69^. EBA-175 and PvMSP1 had near-atomic accuracy resolutions (<2.5 Å) because their structures were determined by high-accuracy methods: crystallography and solution NMR^35,70,71^. For PvMSP1, we chose 1 of the multiple experimentally determined conformations.

The AlphaFold 3 generated VAR2CSA (FCR3) core region was evaluated against cryo-EM resolved structures for the same area (**Fig S4**). Relative to the pruned RMSD score, these structures were sufficiently similar to one another (RMSD< 3 Å). Low resolution and a high proportion of unstructured regions may have resulted in a higher whole RMSD score (5.894 Å)^72,73^. Relative to our protein segment of greater than 1400 amino acids, the whole RMSD score suggests high accuracy^74^.
